# Extreme risk protection orders in King County, Washington: the epidemiology of dangerous behaviors and an intervention response

**DOI:** 10.1186/s40621-020-00270-1

**Published:** 2020-07-22

**Authors:** Shannon Frattaroli, Elise Omaki, Amy Molocznik, Adelyn Allchin, Renee Hopkins, Sandra Shanahan, Anne Levinson

**Affiliations:** 1 The Johns Hopkins Bloomberg School of Public Health, Center for Gun Policy and Research, 624 North Broadway, Baltimore, MD 21205 USA; 2 The Johns Hopkins Bloomberg School of Public Health, Department of Health Policy and Management, 624 North Broadway, Baltimore, MD 21205 USA; 3 Educational Fund to Stop Gun Violence, 805 15th Street NW, Washington, DC, 20005 USA; 4 Alliance for Gun Responsibility, P.O. Box 4187, Seattle, WA 98194 USA; 5 Regional Domestic Violence Firearms Enforcement Unit, King County Prosecuting Attorney’s Office, 516 Third Avenue, Room E400, Seattle, WA 98104 USA; 6 Judge (Ret.), Seattle, WA USA

**Keywords:** Firearm injury, Extreme risk protection order, Policy, Suicide, Homicide, Implementation

## Abstract

**Background:**

Extreme Risk Protection Order (ERPO) laws are a promising gun violence prevention strategy. ERPO laws allow specific categories of people (law enforcement in all states, family in most) to petition a court to request that an individual be temporarily prohibited from purchasing and possessing firearms because that individual is behaving dangerously and at risk of violence, either to themselves or others. In 2017 Washington State’s ERPO law took effect. King County developed a comprehensive approach to implementing the ERPO law. The early experience of King County offers important insight into how early adopters of these laws are incorporating EPROs into their approach to gun violence prevention.

**Methods:**

We systematically reviewed, abstracted and coded data from every ERPO petition filed in King County in 2017 and 2018, and all ERPO court records associated with those petitions. We conducted descriptive analyses of the coded data.

**Results:**

Seventy-five ERPO petitions were filed in King County during the study period. Judges granted a temporary ERPO in all 75 cases; 65 (87%) of these cases resulted in a one-year ERPO. Law enforcement initiated 73 (97%) of these petitions, and family members filed the remaining two. The 75 petitions filed described respondents’ risk as to “themselves only” in 30 cases (40%), to “others only” in 20 cases (27%) and “to themselves and others” in 25 cases (33%). Five cases where the threat was to “others only” met a definition of mass shooting threat. For 95% of the temporary ERPOs issued, the courts’ reasoning for issuing ERPOs included either current violence or brandishing a firearm. Court records for the 75 cases detail firearms removed and/or include receipts for removed firearms in 61 cases (81%) either as part of ERPO precipitating events (*n* = 13, 17%) or in conjunction with ERPO service (*n* = 48, 64%).

**Conclusions:**

These findings suggest that Washington’s ERPO law is being applied when someone is threatening violence to self or others, or brandishing a gun and at least one other risk factor is present. The early experience of King County provides insight into how this law is being implemented in one jurisdiction and how courts are evaluating such cases.

## Introduction

As of July 1, 2020, 19 states and the District of Columbia (DC) authorize specific categories of people (e.g., law enforcement, family, household members) to petition a court for a civil order to temporarily restrict persons at extreme risk of harming themselves or others from having access to guns, by prohibiting gun purchase and possession. (Bloomberg American Health Initiative, n.d.) These orders are generally known as Extreme Risk Protection Orders (ERPOs), although not all statutes use this term. For example, California uses the term Gun Violence Restraining Order (GVRO), Risk Protection Order (RPO) is the name used under Florida law, and Lethal Violence Protection Order (LVPO) describes orders in Delaware. (Bloomberg American Health Initiative, n.d.) Media and policymakers commonly refer to these laws as red flag laws. For simplicity (and because the term "red flag law" stigmatizes individuals with mental illness and mischaracterizes the evidence-based approach these laws use), we refer to these orders as ERPOs. All but 2 of these ERPO laws were adopted since 2016, based on a recommendation by the Consortium for Risk-based Firearm Policy released in the aftermath of the Sandy Hook Elementary School shooting in Connecticut. (Consortium for Risk-Based Firearm Policy 2013; McGinty et al. 2014) Typically, legislatures have passed ERPO laws in response to mass shootings. While a case series using California data documents that individuals threatening mass violence are respondents to these orders, (Wintemute et al. 2019) both early commentary (Frattaroli et al. 2015) and recent studies (Kivisto and Phalen 2018; Swanson et al. 2019; Swanson et al. 2017) suggest that ERPO laws are also a promising strategy for preventing suicide. We do note that these outcome studies to date reflect findings from Connecticut and Indiana, the two states with early “risk warrant” laws that predate the Consortium's recommendation and which differ in some procedural ways from that recommendation such as who can initiate the formal process (only law enforcement can request a risk warrant, although Florida, New Mexico, Rhode Island, and Virginia -- some of the most recent states to enact ERPO laws -- also limit petitioners to law enforcement) and the court proceedings that follow. (Bloomberg American Health Initiative n.d.) The rapid uptake of ERPO laws across the United States provides an opportunity to answer questions about how ERPOs are being used (e.g., in response to threats of suicide, violence against others and/or mass shootings), inform implementation policy and practice, and provide a foundation for future ERPO evaluations.

We sought to explore ERPO implementation by examining the experience of King County, Washington. We chose to focus on King County for several reasons. King County’s ERPO implementation processes built on the regional effort there to strengthen policies and practices to enforce civil court orders prohibiting domestic violence offenders from purchasing and possessing firearms. Leaders in King County, the most populous county in the state and home to Seattle, established the Regional Domestic Violence Firearms Enforcement Unit (Unit), composed of police, prosecutors, a court orders “problem-solver,” and victim advocates with expertise in intimate partner violence and gun laws. Premised on a risk-based, public health approach to reduce the risk of harm,  improving swift and certain compliance with firearm dispossession orders for respondents to Domestic Violence Protection Orders (DVPOs) (who are prohibited from accessing, purchasing and possessing firearms and other dangerous weapons, or having a concealed pistol license while subject to a DVPO) is a priority of the Unit. Because ERPO laws follow a similar process that is based on state DVPO laws, (Frattaroli et al. 2015) the basic infrastructure needed to implement ERPOs was already in place. In addition, the Unit worked with the Seattle Police Department’s Crisis Response Unit – which responds to people experiencing a behavioral crisis (City of Seattle 2019) – to assure both their experience and their personnel were part of ERPO implementation. Washington was the second state, after California, of the 17 states that enacted an ERPO law post-Sandy Hook Elementary School shooting, and King County, in particular, through its new regional Unit, was an early ERPO adopter. As a result, the County’s initial efforts yielded sufficient quantities of ERPOs to inform this initial analysis. Finally, we view the experience of King County, and the Unit, as informative for ERPO implementation in other jurisdictions.

### Overview of Washington law

Washington voters passed ballot initiative 1491 (that would become the State’s ERPO law) on November 8, 2016 with 69% of voters statewide and a majority of voters in 32 of the State’s 39 counties, including King County. (Ballotpedia 2016) The law took effect on December 8, 2016. Washington’s ERPO law authorizes law enforcement, family (including intimate partners), and household members to first petition a court for a temporary (“*ex parte”)* ERPO to be issued without notice to the respondent when petitioners “have personal knowledge that the respondent poses a significant danger of causing personal injury to self or others in the near future by having in his or her custody or control, purchasing, possessing, or receiving a firearm.” (Revised Code of Washington Chapter 7.94 n.d.) These orders are emergency orders that may be issued by a court when the threat of harm is imminent.

The petition for the temporary order results in an immediate hearing that includes the petitioner and judge who grants or denies the request for a temporary ERPO based on whether the information provided by the petitioner, under penalty of perjury, establishes reasonable cause to enter the order. The petition must identify the number, types, and locations of any firearms the petitioner believes to be in the respondent’s possession, if any, so that law enforcement can remove them when the order is served. To determine a respondent’s level of risk, courts are to consider certain factors that research has demonstrated indicates a person is at an elevated risk of committing harm or violence to themselves or others, which are specified in the law (e.g., violent act or threat, violation of a protection order, problematic substance use, criminal history, brandishing a firearm, access to or intention to acquire a firearm).

The court must then hold a hearing within 14 days to determine if a one-year ERPO should be issued. The respondent must be served with notice of the hearing, including the underlying petition and temporary order, at least five court days prior to the full hearing. During the hearing, both the petitioner and the respondent participate and the judge determines whether a preponderance of evidence has been established to issue an ERPO. Both temporary and one-year EPROs prohibit respondents from having custody or control, purchasing or attempting to purchase, possessing or receiving, firearms, or having a concealed pistol license, for the duration of the order.

In 2019, Washington amended its law to replace “dangerous mental health issues” with “behaviors that present an imminent threat of harm to self or others,” add hate crime convictions as criteria for courts to consider when issuing an ERPO, explicitly include minors as ERPO respondents, make the court process more immediate for officers dealing with rapidly emergent threats, and clarify other aspects of the law. (Washington State Legislature 2019).

While the literature documents how some local jurisdictions implement DVPOs, (Frattaroli and Teret 2006; Wintemute et al. 2014) there is scant information about ERPO implementation. In one evaluation of Connecticut’s risk warrant law (an ERPO-style law enacted in 1999 that allows only for law enforcement to petition the court) the authors include perspectives from law enforcement about implementation processes. (Swanson et al. 2017) Here we seek to add to the Connecticut and Indiana findings, and to the California case series, by addressing three questions: Who are the petitioners and respondents in ERPO cases? What are the behaviors described in ERPO petitions? How have courts responded to ERPO petitions?

## Methods

We reviewed all ERPO petitions filed and heard in King County municipal, district or superior courts from January 1, 2017 through December 31, 2018. Case documents included the petitions and supporting materials, temporary ERPOs and one-year ERPOs issued, receipts for firearms obtained, and respondent and petitioner information provided to the court. Because these are publicly available data, our institutional review board deemed this study to be "not human subjects research".

We abstracted data from these court records using a single form developed by the research team. We used an early version of the form to review a small number of cases and made minor modifications to clarify and streamline the data collection process. The final form captured data about respondent demographics, whether the petitioner was a law enforcement officer or a family/household member, respondent criminal history (if any), and narrative text detailing the petitioner’s encounter or past interactions with the respondent that preceded the ERPO petition. We also included information about the court’s basis for issuing an ERPO, as selected from a list of options on the ERPO form. Washington’s ERPO law requires the court to make findings regarding the grounds for issuing an ERPO. If the court declines to issue an ERPO, it must include in the record the particular reason(s) for the court’s denial. Between February and April 2019 we abstracted data from an internal case management system maintained by the King County Prosecuting Attorney’s Office (where the Unit is located) about the number of firearms removed from each respondent after the court issued an ERPO and entered all resulting data into a Microsoft Access database. We used SAS 9.4 for all analyses.

Our team a-priori identified summary variables of interest informed by our research questions and guided by the data abstraction form. We also sought to identify cases where mass shooting was a risk, and used the definition developed by Wintemute, et al. to identify such cases. That definition includes two parts: “1) a judicial officer issued [an ERPO] after the subject of the order had made a clear declaration of intent to commit a mass shooting or had exhibited behavior suggesting such an intent; and 2) the subject had or would soon have access to firearms.” (Wintemute et al. 2019) After receiving an introduction to the court records system, two authors (EO and AM) independently coded all of the narratives and then met to discuss and reconcile any disagreements. When the two coders were unable to agree, a third coder (SF) served as the tiebreaker. The two main coders, who were new to reviewing court records, had ready access to a representative from the prosecutor's office (co-author SS) where they accessed the ERPO records and participated in regular study team meetings with the representative and a retired judge (co-author AL).

Resulting analyses include descriptive statistics of respondent demographics, categories of petitioners, and petition characteristics. Using the coded risk status variables, we generated a mutually exclusive analytical variable for risk to self only, risk to others only, or risk to both self and others.

## Results

Here we describe the incidents that precipitated the ERPO petitions, the courts’ decisions about those petitions at each hearing, and the firearms removed in response to those court orders.

### ERPO petitioners and respondents

In 2017 there were 3 ERPO petitions filed in King County, Washington; in 2018, there were 72. Respondents were primarily male and white (Table 1) with an average age of 42; most (*n* = 43, 57%) respondents had multiple law enforcement encounters noted in the court records (not included in the tables) prior to the incident leading to the ERPO petition noted in the court records. Relative to the King County population, (US Census Bureau 2020) ERPO respondents were more often male (84% vs. 50%), and white (75% vs. 67%) or black (12% vs. 7%). Of the 75 petitions, family and partners were the petitioners for two. The remaining 73-law enforcement-initiated petitions resulted from calls for assistance most often initiated by family or friends of the respondents (*n* = 38, 51%) or the respondents themselves (*n* = 13, 17%). Among the family or friends who called law enforcement, most (84%) were eligible petitioners under the law because of their familial relationship to the respondent. Whether they were aware of ERPOs when they called law enforcement or knew they were eligible petitioners, is not consistently noted in the court records. The remainder of the calls to law enforcement came from other groups, as noted in Table 1.

**Table 1:** ERPO respondents and petitioners

**Descriptor**	**N (%)**
Respondent Mean, Median Age (SD)	41.6, 37.5 (16.3)
Respondent Sex	
Male	63 (84%)
Female	12 (16%)
Respondent Race	
White	56 (75%)
Black	9 (12%)
Asian	4 (5%)
Other	2 (3%)
Unknown	4 (5%)
Petitioner	
Seattle Police Department	46 (61%)
Other King County Law Enforcement Agency	27 (36%)
Family Member of Respondent	2 (3%)
Petitioner was notified by:	
Friend or family member	38 (51%)
Eligible Petitioner	32 (43%)
Non-Eligible Petitioner	6 (8%)
Respondent	13 (17%)
Members of the Public	9 (12%)
Neighbors	6(8%)
Health Care Agency	4 (5%)
Law Enforcement Agency	1(1%)
Other or Unknown	4 (5%)

### Dangerous behaviors that precipitated ERPO petitions

The 75 petitions filed described respondents’ risk as to “themselves only” in 30 cases (40%), to “others only” in 20 cases (27%) and “to themselves and others” in 25 cases (33%) (Table 2.) The three most common dangerous behaviors we coded, based on descriptions of events that precipitated the ERPO filings noted in the court records, varied across these three risk profiles. Among the 30 respondents posing a risk to themselves only, each of their court records included a current, explicit expressed desire, intention or action to end their life; 14 (47%) noted substance use that caused concern among those on the scene of the precipitating event; and eight (27%) included brandishing a firearm. For the 20 respondents deemed to be a risk to others only, ERPO court documents for 18 (90%) cases included threats or acts of violence; 12 (60%) included reports of brandishing a firearm; and 10 (50%) had a history of law enforcement encounters (arrest only, or arrest with conviction). The 25 respondents posing a risk to both themselves and others had on average the highest number of dangerous behaviors coded, with court records noting a current explicit expression to end their life among most (*n* = 16, 64%); substance use that caused concern among those on the scene in 15 (60%) cases; and 14 (56%) with past law enforcement criminal encounters (Table 2). While family members and friends who initiated the calls for assistance that preceded ERPO petitions most often (*n* = 18, 47%) reported respondent self-harm risks, respondents who initiated calls for assistance were almost equally at risk of self-harm (*n* = 5, 38%), harm to others (*n* = 4, 31%) and both (n = 4, 31%). Those at risk of suicide self-reported directly to law enforcement, indirectly through a crisis hotline in three cases, or expressed suicidal intent while being arrested for driving under the influence. The other eight respondents who self-reported contacted law enforcement with complaints about others’ risky behaviors (n=3), or threatening to harm others (n=5).

**Table 2:** Petitioners’ dangerous behaviors noted in court records

**Risk Factor**	**Risk to Self only** ***N*= 30 (40%)**	**Risk to Others only** ***N*= 20 (27%)**	**Risk to Self and Others** ***N*= 25 (33%)**	**Total** ***N*= 75**
Suicide risk				
Yes	30 (100%)	0	25 (100%)	55 (73%)
Explicit behaviors	30 (100%)	0	16 (64%)	46 (61%)
Implicit behaviors	0	0	9 (36%)	9 (12%)
No or Unknown	0	20 (100%)	0 (0%)	20 (27%)
Risky Substance Use				
Yes, Substance(s):	14 (47%)	6 (30%)	15 (60%)	35 (47%)
Alcohol	13 (43%)	5 (25%)	10 (40%)	28 (37%)
Marijuana	1 (3%)	3 (15%)	5 (20%)	9 (12%)
Prescription Drugs	2 (6%)	0 (0%)	4 (16%)	6 (8%)
Opioids	1 (3%)	1 (5%)	1 (4%)	3 (4%)
Illicit Drugs	0 (0%)	1 (5%)	2 (8%)	3 (4%)
Other/Unknown	2 (7%)	1 (5%)	3 (12%)	6 (8%)
No or Unknown	16 (53%)	14 (70%)	10 (40%)	40 (53%)
Threatening violence or violent behaviors				
Yes	3 (10%)	18 (90%)	14 (56%)	35 (47%)
No or Unknown	27 (90%)	2 (10%)	11 (44%)	40 (53%)
Criminal History				
Yes	4 (13%)	10 (50%)	14 (56%)	28 (37%)
No or Unknown	26 (87%)	10 (50%)	11 (44%)	47 (63%)
Brandished firearm				
Yes	8 (27%)	12 (60%)	7 (28%)	27 (36%)
No or Unknown	22 (73%)	8 (40%)	18 (72%)	48 (64%)
Irrational/erratic behaviors				
Yes	2 (7%)	9 (45%)	12 (48%)	23 (31%)
No or Unknown	28 (93%)	11 (55%)	13 (52%)	52 (69%)
History of domestic violence				
Yes	3 (10%)	8 (40%)	5 (20%)	16 (21%)
No or Unknown	27 (90%)	12 (60%)	20 (80%)	59 (79%)
Expressed intent to obtain firearm				
Yes	4 (13%)	2 (10%)	2 (8%)	8 (11%)
No or Unknown	26 (87%)	18 (90%)	23 (92%)	67 (89%)
Recently purchased firearm				
Yes	3 (10%)	0 (0%)	2 (8%)	5 (7%)
No or Unknown	27 (90%)	20 (100%)	23 (92%)	70 (93%)
Number of risk factors identified through coding court files				
Mean (SD)	2.37 (1.16)	3.25 (0.91)	3.48 (0.96)	2.97 (1.14)
Median (Range)	2 (1–6)	3 (2–5)	4 (1–5)	3 (1–6)

Five (7%) of these cases met Wintemute’s definition of mass shooting threat. (Wintemute et al. 2019) All of these respondents threatened to shoot and kill specific groups (i.e. law enforcement, school communities) and two respondents additionally threatened violence against the public generally. In two cases, the threatened settings were evacuated in response to the threats. Court records note that all of these respondents expressed an intention to buy firearms. There are no references to any of these respondents already possessing firearms prior to the petition or to law enforcement removing firearms as part of ERPO service, indicating one or more other required factors were present that warranted a prohibition on purchase and future possession nonetheless.

### Court decisions on ERPO petitions

Courts issued temporary ERPOs for all 75 petitions. The three most frequent reasonable cause findings courts cited for granting temporary ERPOs in response to these petitions were violent threats and/or behaviors (*n* = 62, 86%), a pattern of violence (*n* = 34, 47%), and brandishing a firearm (*n* = 32, 44%); 71 of the 75 (95%) cases had at least one of these findings (Table 3). In most cases (*n* = 69, 92%), judges selected multiple findings. While these findings varied somewhat among those petitioners identified as “at risk of suicide only,” “risk to others only,” or “risk to both self and others,” violent threats and/or behaviors was the leading finding for all three categories (Table 3).

**Table 3:** Courts’ reasonable cause findings for issued temporary ERPOs

**Reasonable Cause for temporary ERPO**^**2**^	**Risk to Self only** ***N*= 29 (40%)**	**Risk to Others only** ***N*= 19 (27%)**	**Risk to Self and Others** ***N*= 24 (33%)**	**Total** ***N*= 72**^**1**^
a. Violence and threats	28 (97%)	15 (79%)	19 (79%)	62 (86%)
b. Pattern of violence	8 (28%)	13 (68%)	13 (54%)	34 (47%)
c. Brandished	11 (38%)	12 (63%)	9 (38%)	32 (44%)
d. Dangerous Mental Health Issues^3^	10 (34%)	6 (32%)	13 (54%)	29 (40%)
e. Substance Use	8 (28%)	5 (26%)	6 (25%)	19 (26%)
f. Intent to obtain	7 (24%)	2 (11%)	6 (25%)	15 (21%)
g. Felony Crime	0 (0%)	6 (32%)	8 (33%)	14 (19%)
h. Force	1 (3%)	7 (37%)	6 (25%)	14 (19%)
i. DV Crime	0 (0%)	6 (32%)	7 (29%)	13 (18%)
j. Access	7 (24%)	2 (11%)	3 (13%)	12 (17%)
k. Recently Acquired	5 (17%)	2 (11%)	3 (13%)	10 (14%)
l. Violated PO	0 (0%)	2 (11%)	1 (4%)	3 (4%)
m. Stalking	0 (0%)	3 (16%)	0 (0%)	3 (4%)
n. Other	1 (3%)	3 (16%)	1 (4%)	5 (7%)
**Petition Outcome**	**Risk to Self only** ***N*= 30 (40%)**	**Risk to Others only** ***N*= 20 (27%)**	**Risk to Self and Others** ***N*= 25 (33%)**	**Total** ***N*= 75**
Temp ERPO Granted	30 (100%)	20 (100%)	25 (100%)	75 (100%)
Full ERPO Granted	26 (87%)	18 (90%)	21 (84%)	65 (86%)
Full ERPO Denied	4 (13%)	2 (10%)	2 (8%)	8 (11%)
Case Dismissed	0 (0%)	0 (0%)	2 (8%)	2 (3%)

^1^In three cases (one each from risk to self, risk to others, and risk to self and others), the temporary ERPO was issued, but no reasonable cause was selected

^2^Reasonable causes for ERPO are:

a. Violence: Respondent has recently committed or threatened violence against self or others, whether or not respondent had a firearm

b. Pattern: Respondent has shown, within the past 12 months, a pattern of acts or threats of violence, which can include violent acts against self or others

c. Brandished: Respondent has unlawfully or recklessly used, displayed, or brandished a firearm

d. Dangerous mental health issues: Behaviors that present an imminent threat of harm to self or others

e. Substance use: There is corroborative evidence of the respondent’s abuse of alcohol or controlled substances

f. Intent to obtain: Respondent expressed intent to obtain a firearm(s)

g. Felony crime: Respondent has been arrested for or convicted of a felony offence or violent crime

h. Force: Respondent has a history of use, attempted use, or threatened use of physical force against another person

i. DV crime: Respondent has been arrested for or convicted of a domestic violence crime

j. Access: Respondent has access to someone else’s firearms

k. Recently aquired: Respondent recently acquired a firearm

l. Violated PO: Respondent violated a civil or criminal protection order, no-contact order or restraining order. m. Stalking: Respondent has a history of stalking another person

n. Other

^3^In 2019, Washington amended its law to replace “dangerous mental health issues” with “behaviors that present an imminent threat of harm to self or others”

Of the 75 temporary orders issued, court records indicate that 14 respondents appeared at the hearing to contest the issuance of a one-year ERPO. In eight of these cases, the court denied the requested order (finding that the legal standard for the court to issue a one-year order was not met). The court dismissed an additional two cases: one after the respondent died in a motor vehicle crash and a second because the respondent was prohibited from possessing firearms through a felony conviction resulting from the same incident that initiated the ERPO petition. Courts granted ERPOs in the remaining four contested cases. Among the denied petitions, all eight had included a reasonable cause finding of violent threats or acts (directed at self and/or others) for the temporary ERPO and in five of the eight cases the court continued the temporary ERPO at least once (mean length of temporary ERPOs continued in these five cases = 129 days, range 28–407 days) so that it would remain in place during any delays surrounding the hearing date. This small number of cases resulting in denied and dismissed ERPOs precludes significance testing of comparisons with the cases for which courts granted ERPOs.

### Impacts of ERPO petitions: firearms removed

Among the 75 cases, court records detail firearms removed and/or include receipts for removed firearms in 61 cases (81%) either as part of ERPO precipitating events (*n* = 13, 17%) or in conjunction with ERPO service (*n* = 48, 64%). For 10 cases (13%) the descriptions of respondents’ dangerous behaviors that prompted the ERPO petitions did not include information about respondents possessing firearms. The records do note in seven of those 10 cases that respondents expressed their intention to obtain firearms, but in three cases respondents neither possessed nor expressed plans to obtain firearms. In these cases, court records include declarations from the respondents as documentation that the respondents did not have firearms to relinquish. The remaining four cases (5%) include information about respondents’ firearms, but there is no indication that the respondents were dispossessed of those firearms after the court issued ERPOs.

## Discussion

This descriptive analysis of the initial years of ERPO implementation in King County provides early insight into how these laws are being used to intervene when people are behaving dangerously and at risk of committing gun violence and informs what policymakers, implementers, and the public can expect from ERPO laws.

### Who are the petitioners and respondents in ERPO cases?

Law enforcement filed the overwhelming majority (97%) of ERPO petitions in King County. Of note, family members were most frequently the group to engage law enforcement in the events that precipitated ERPO petitions (43%), followed by respondents (17%). The role of family and the eventual respondent in engaging with law enforcement suggests that at times family and individuals at risk are well-positioned to recognize when the risk of violence is escalating and law enforcement intervention is needed. Whether family who called for law enforcement assistance in these cases were aware of ERPOs or seeking law enforcement assistance with initiating an ERPO is not known based on these data. Whether family members seeking law enforcement assistance would petition on their own and under what circumstances, are questions worth exploring as states and localities assess how best to implement ERPO laws.

ERPO respondents in King County were overwhelmingly male (84%) with an average age of 42. Both white and black respondents are over-represented in the King County data. As ERPO implementation evolves in communities throughout the country, we urge implementers and researchers to understand how ERPOs are being used, particularly with communities of color where relationships with law enforcement are too often strained. ERPOs are an upstream civil intervention with few collateral consequences relative to traditional criminal justice options (e.g., arrest, incarceration, involuntary treatment), so for those reasons it may be seen as a preferred approach. Even so, assuring ERPO implementation is transparent and constructive, and does not result in unjust punitive consequences, will be important to realizing the preventive intention -- minimizing risk of harm due to firearm violence -- and potential of this law. Engaging with communities about how to use ERPOs effectively is an investment worth making. That most of the King County petitions resulted from contact initiated by family, friends and the respondents themselves suggests that law enforcement is using ERPOs as a tool to help respond to calls for assistance as one of several intervention options, and not as an enforcement action to target particular groups.

### What are the behaviors described in ERPO petitions?

The largest segment of petitions filed was in response to suicide risk (40%) and risk of harm to self and others (33%). Fully 73% of respondents threatened self-harm. Those threatening harm to others only were the smallest group of ERPO respondents in King County but included all five mass shooting threats we identified. The mass shooting threats represent 7% of all temporary ERPOs issued, which is less than the 13% reported by Wintemute and colleagues in their analysis of California data. (Wintemute et al. 2019) With the inclusion of hate crimes in the 2019 amendments to Washington’s ERPO law, use of ERPOs in response to mass shooting threats may increase.

### How have courts responded to ERPO petitions?

King County ERPO petitions consistently met the legal standard set forth in Washington law as evidenced by the courts’ decisions to grant temporary ERPOs for every petition filed, and to grant full ERPOs in 87% of those cases. The petitions described people who were expressing their intentions to commit violence, and most possessed firearms as well. While those narratives included many indicators of risk (risky substance use, criminal histories, and access or intention to obtain firearms) for 95% of the temporary ERPOs issued, the courts’ reasoning for issuing ERPOs included either current violence or brandishing a firearm. These findings suggest that Washington’s ERPO law, which defines ERPOs for use when an individual “poses a significant danger of causing personal injury to self or others in the near future by having in his or her custody or control, purchasing, possessing, or receiving a firearm” is being applied when someone is threatening violence to self or others, or brandishing a gun and at least one other risk factor is present. We did not observe ERPO petitions filed or issued solely because of risky substance use, involvement with the justice system, or access to a gun. Rather, these facts appear to be secondary to violent threats and actions when petitioners and the courts assess whether an ERPO is an appropriate response.

When respondents contested ERPOs in King County, the court tended to deny the one-year ERPO. Due to the small number (*n* = 8) of cases the court denied at the one-year order hearing, we were unable to test for any differences between contested orders that were granted and those that were denied. However, we do note that the denied cases all involved threats or acts of violence. Furthermore, from the perspective of the reviewers on our team, denied cases appeared qualitatively similar to cases where one-year ERPOs were issued in terms of the level of dangerousness conveyed through the court documents. Whether respondents who successfully contest are different from those whose cases result in a full ERPO in terms of their risk, representation by counsel or other factors, and whether petitions that are ultimately denied (and thus shorten the time that a respondent is dispossessed of firearms) affect personal or public safety are all empirical questions that should be pursued.

### Do ERPOs result in firearm removal?

When courts in King County issue an ERPO, most often respondents are dispossessed of firearms. Whether dispossession happens at the precipitating event, during service of the order, or is initiated by the respondent, the infrastructure in place in King County is resulting in firearms removed. Importantly, ERPOs in Washington are also being used to preempt firearm acquisition when an individual is at heightened risk of committing violence. This is consistent with both the intent of ERPOs and Washington law. (Revised Code of Washington 7.94 n.d.) The five mass shooting threats included in the data were all instances where the threat was real, even though the respondents did not have a firearm that law enforcement was aware of.

Our findings are largely consistent with early research from Connecticut and Indiana about use of ERPO laws. (Swanson et al. 2017; Swanson et al. 2019) Temporarily removing firearms from people who are in crisis was demonstrated to be feasible in Connecticut and Indiana, and the demographic profile of respondents is similar across the data in Indiana, Connecticut and King County. The largest segment of petitions in all three jurisdictions was in response to suicide risk (61 and 68% in Connecticut and Indiana, respectively). Likewise, police were informed about the respondent by family members in Connecticut and Indiana at similar proportions as in King County. Unlike in King County, however, neither Connecticut nor Indiana include any cases where the eventual respondent initiated the law enforcement contact that would lead to an ERPO petition.

### Limitations and strengths

Here we present a descriptive study of ERPO petitions from one jurisdiction served by several law enforcement agencies, based on a review of court records. As such, the findings presented herein offer insight into the experience of one county and are limited to information compiled through the courts. Future research is needed to better understand other localities’ experiences with ERPOs, and how they operate in the context of their state firearms laws. Additional data, such as perspectives of petitioners and respondents, would also add to these findings. While the single county focus limits the generalizability of these findings, the level of detail from this first insight into a complete set of EPRO data from a locality that is leading implementation efforts on this topic is instructive. We include all cases in the study and provide a detailed description of how this law is being used. Our findings are based on a rigorous process of double-coding and informed by both researchers and practitioners who together provide a comprehensive understanding of ERPO law, and its implementation.

## Conclusion

At a time when policymakers are considering ERPO laws in state legislatures around the country, and states as well as local jurisdictions are developing protocols and practices to implement ERPOs, the early experience of King County provides insight into how this law is being used in one jurisdiction and how courts are evaluating cases.

## Data Availability

The data used in the analyses presented herein are publicly available.
